# Does rate of weight regain predict premature discontinuation of treatment during outpatient cognitive-behavioural therapy for eating disorders?

**DOI:** 10.1186/2050-2974-3-S1-O43

**Published:** 2015-11-23

**Authors:** Amy Lampard, Olivia Carter, David Erceg-Hurn, Bronwyn Raykos, Anthea Fursland

**Affiliations:** 1 School of Psychology and Speech Pathology, Curtin University, Australia; 2 Centre for Clinical Interventions, Australia

## 

Between 20-51% of eating disorder clients in inpatient settings and 29-73% in outpatient settings prematurely discontinue treatment. Research has attempted to address this problem by identifying the client characteristics upon entering treatment that predict dropout, but this approach has failed to identify consistent predictors of treatment dropout. Processes that occur early in treatment, such as early weight gain, may be important to consider as predictors of treatment dropout. Weight restoration is a primary goal of treatment for anorexia nervosa and may also be important in patients with bulimia nervosa who are weight suppressed. Previous research has explored the optimal rate of weight gain, but to our knowledge, no research has considered rate of weight gain as a predictor of risk for treatment dropout. Using weekly assessments of body mass index, the current research therefore aimed to identify whether a rapid rate of weight gain in the early stages of outpatient cognitive-behavioural therapy for eating disorders (CBT-E) is associated with a greater risk of treatment dropout. Participants were adult clients who entered CBT-E treatment for an eating disorder at an outpatient mental health service in Perth, Western Australia. Results will contribute to knowledge of early indicators of risk for treatment dropout.

